# Zn- and Cu-Doped MnFe_2_O_4_ Nanofertilizer: Synthesis, Characterization, and Their Role in Enhancing Fenugreek (*Trigonella foenum-graecum*) Growth

**DOI:** 10.3390/nano16070392

**Published:** 2026-03-24

**Authors:** Dipali R. Ingavale, Vithoba L. Patil, Chaitany Jayprakash Raorane, Sagar M. Mane, Panditrao D. Shiragave

**Affiliations:** 1 Department of Agrochemicals and Pest Management, Devchand College, Kolhapur 416216, Maharashtra, India; deepaliingavale27@gmail.com; 2 Department of Agrochemicals and Pest Management, Shivaji University, Kolhapur 416004, Maharashtra, India; 3 Department of Physics, R. B. Madhkholkar Mahavidyalay, Chandgad 416509, Maharashtra, India; 4 School of Chemical Engineering, Yeungnam University, 280 Daehak-Ro, Gyeongsan 38541, Republic of Korea; 5 Department of Fiber System Engineering, Yeungnam University, 280 Daehak-Ro, Gyeongsan 38541, Republic of Korea

**Keywords:** Zn nutrition, Cu nutrition, spinel ferrite nanoparticles, fenugreek, nano-enabled fertilization

## Abstract

Micronutrient deficiencies and low nutrient-use efficiency remain critical constraints to sustainable crop production. This study tested the hypothesis that Zn- and Cu-doped MnFe_2_O_4_ spinel ferrite nanoparticles can function as an efficient multinutrient nanofertilizer to enhance fenugreek (*Trigonella foenum-graecum* L.) growth and physiological performance. Zn- and Cu-doped MnFe_2_O_4_ nanoparticles were synthesized via a sol–gel method and characterized using X-ray diffraction (XRD), scanning electron microscopy (SEM), and energy-dispersive X-ray spectroscopy (EDS). The nanoparticles exhibited a cubic spinel structure with an average crystallite size of 27 nm and uniform incorporation of Zn and Cu within the MnFe_2_O_4_ lattice. Foliar application at different concentrations (100–500 mg/L) significantly improved seed germination, seed vigor, plant height, leaf number, stem thickness, biomass accumulation, and chlorophyll content compared with the untreated control. The 300 mg/L treatment consistently produced the greatest improvements, increasing plant height, biomass, and total chlorophyll content by more than 25–40% relative to control plants. Higher concentrations of T_5_ resulted in diminished benefits, indicating a concentration-dependent response. These findings demonstrate that Zn- and Cu-doped MnFe_2_O_4_ nanofertilizer provides a balanced and bioavailable source of essential micronutrients, offering a promising nano-enabled strategy for improving nutrient use efficiency and sustainable fenugreek production.

## 1. Introduction

Agriculture remains a cornerstone of the Indian economy, supporting nearly half of the population and playing a critical role in food security and rural livelihoods, thereby necessitating sustainable strategies to enhance crop productivity while minimizing environmental impacts [1]. Conventional agricultural intensification, particularly since the mid 20th century, has relied heavily on chemical fertilizers to improve crop yields; however, excessive and inefficient fertilizer use has resulted in nutrient losses, soil degradation, and ecological concerns [2]. Fertilizers, whether natural or synthetic, are essential inputs that supply nutrients required for optimal plant growth and enhanced crop productivity. They restore soil fertility by replenishing nutrients removed by previous cropping cycles [3]. Plants require at least sixteen essential elements for proper growth. Among these, carbon, hydrogen, oxygen, nitrogen, phosphorus, potassium, sodium, calcium, and magnesium are classified as macronutrients, while zinc, copper, iron, molybdenum, boron, manganese, and silicon constitute the micronutrient group [4]. In recent years, nanotechnology has emerged as a transformative tool in modern agriculture, attracting considerable attention from researchers and farmers alike. Its potential to contribute to sustainable agricultural practices is increasingly recognized [5]. Key applications of nanotechnology in agriculture include the development of nano fertilizers, nano pesticides, nanosensors, and technologies for soil remediation [6]. Nanoparticles, typically ranging in size from 1 to 100 nm, possess unique physicochemical properties distinct from their bulk counterparts [7].

Nanofertilizers are designed to ensure a controlled and sustained release of nutrients, thereby improving nutrient availability, minimizing losses, and enhancing overall soil productivity. Their high surface area promotes efficient nutrient uptake, increased photosynthetic activity, and greater biomass accumulation, while also contributing to improved plant tolerance under abiotic stress [8]. Micronutrients such as zinc (Zn), copper (Cu), iron (Fe), and manganese (Mn) play vital roles in numerous physiological and biochemical processes in plants, ultimately enhancing crop growth and yield. Nano-micronutrient fertilizers, enriched with essential elements like Fe, Mn, Zn, Cu, B, Mo, and Ni, have shown promise in improving both yield and nutritional quality through enhanced nutrient-use efficiency [9]. Several studies have demonstrated the effectiveness of nano-based fertilizers in agricultural systems. ZnO nanoparticles have been shown to function as efficient zinc nanofertilizers for rice [10], supported by their distinctive optical, physical, and antimicrobial properties [11]. Nano-copper supplementation has yielded significant improvements in wheat growth [12], with copper nanomaterials exhibiting both insecticidal and fertilizing capabilities, suggesting their potential dual use as nanopesticides and nanofertilizers [13]. Similarly, ferric oxide nanoparticles have been reported to enhance biochemical traits such as starch, total chlorophyll, carotenoids, and phenolics in ginger [14], while nano-iron oxide has been shown to accelerate growth and yield attributes in Glycine max [15] and manganese and iron nanofertilizers have demonstrated beneficial effects on edible plant production [16]. Studies found significant improvements in growth performance of cabbage and lupin following the application of synthesized Zn, Fe, and Mn-based nanofertilizers [17].

Fenugreek (*Trigonella foenum-graecum* L.) is one of the oldest known medicinal plants, belonging to the family Fabaceae, and is believed to have originated in Central Asia around 4000 BC [18]. The seeds of fenugreek are particularly valued for their rich composition, containing substantial amounts of gum, dietary fiber, alkaloids, flavonoids, saponins, and various volatile compounds. Owing to its high fiber content, fenugreek has been utilized in food systems as a natural stabilizing, adhesive, and emulsifying agent, contributing to textural modifications for specialized food applications. Fenugreek seeds and leaves possess a wide range of pharmacological properties and are traditionally used for managing diabetes, hypercholesterolemia, hypoglycemia, and anorexia, as well as for their antioxidant, antibacterial, anticarcinogenic, and gastroprotective effects [19]. The antidiabetic potential of fenugreek seeds has been demonstrated in animal studies [20]. Recognized as one of the earliest medicinal plants, fenugreek continues to be valued for its remarkable nutritional and therapeutic benefits [21].

In this context, the present study reports, for the first time, the synthesis and comprehensive characterization of Zn- and Cu-doped MnFe_2_O_4_ ferrite nanoparticles and systematically investigates their role as multifunctional nano-micronutrient fertilizers for fenugreek growth enhancement. The novelty of this work lies in the use of dual-doped manganese ferrite nanoparticles as a single nanofertilizer system supplying multiple essential micronutrients, the correlation between dopant-induced structural and physicochemical properties and plant growth responses, and the evaluation of their potential to improve sustainable nutrient management in agriculture. This study aims to provide new insights into the design of ferrite-based nanofertilizers for improved crop productivity with reduced nutrient losses.

## 2. Materials and Methods

### 2.1. Materials

Manganese sulfate monohydrate (MnSO_4_·H_2_O, ≥99%), ferrous sulfate heptahydrate (FeSO_4_·7H_2_O, ≥98.5%), copper sulfate pentahydrate (CuSO_4_·5H_2_O, ≥98.5%), zinc sulfate heptahydrate (ZnSO_4_·7H_2_O, ≥99%), and aqueous ammonia solution (30%) were purchased from Qualigens (Thermo Fisher Scientific India Pvt. Ltd., Mumbai, India). All chemicals were of analytical grade and used without further purification. Fenugreek (*Trigonella foenum-graecum* L.) seeds were procured from a certified local agricultural supplier in Sangli district, Maharashtra, India.

### 2.2. Synthesis of Zn- and Cu-Doped MnFe_2_O_4_ Nanostructured Fertilizer

Synthesis of Zn- and Cu-doped MnFe_2_O_4_ nanostructured fertilizer used the sol–gel method, because the sol–gel technique is preferred for producing high-purity materials [22]. Zn- and Cu-doped MnFe_2_O_4_ nanostructured fertilizer was synthesized using a sol–gel method with precisely defined precursor ratios. Manganese sulphate monohydrate (MnSO_4_·H_2_O, 99%), ferrous sulphate heptahydrate (FeSO_4_·7H_2_O, 98.5%), copper sulphate pentahydrate (CuSO_4_·5H_2_O, 98.5%), and zinc sulphate heptahydrate (ZnSO_4_·7H_2_O, 99%) were used as metal ion sources. The precursor solutions were prepared by dissolving 0.3 mol each of MnSO_4_·H_2_O, CuSO_4_·5H_2_O, and ZnSO_4_·7H_2_O and 2.0 mol of FeSO_4_·7H_2_O in deionized water under constant stirring. The overall molar ratio of (Zn + Cu + Mn): Fe was maintained at 1:2, yielding a nominal ferrite composition of (Zn_0.3_Cu_0.3_Mn_0.3_): (Fe_2_). The mixed solution was heated to 70 °C and magnetically stirred to ensure homogeneity. Aqueous ammonia solution (25%) was added dropwise to adjust the pH to 9.0 ± 0.2, resulting in the formation of a uniform solution. The sol was aged for 12 h, followed by drying at 100 °C to obtain a xerogel. The dried precursor was calcined at 500 °C for 3 h in ambient atmosphere to achieve crystallization of the doped manganese ferrite phase. After cooling to room temperature, the calcined product was finely ground and stored for subsequent physicochemical characterization and plant growth experiments.

### 2.3. Characterization

For confirmation of material and to determine particle size, characterization of Zn- and Cu-doped MnFe_2_O_4_ nanoparticles was done by XRD, i.e., X-ray diffraction spectroscopy model AXS D8 Advance (Bruker India Scientific Pvt. Ltd., New Delhi, India). Surface morphology and grain size of the prepared powder were studied using a scanning electron microscope (SEM) equipped with Energy Dispersive X-Ray Analysis EDAX, JEOL, JSM-IT200 (JEOL India Pvt. Ltd., New Delhi, India). Dynamic light scattering (DLS) Malvern Nano ZS 90 ZEN 3696 (Malvern Panalytical, Gurgaon, India) was used for particle size analysis.

### 2.4. Bioassay (Seed Germination) of Nanostructured Zn- and Cu-Doped MnFe_2_O_4_ Fertilizer

Seed germination experiments were conducted in the DST-FIST Research Laboratory, Devchand College, Arjunnagar, Maharashtra, India. Certified seeds of fenugreek (*Trigonella foenum-graecum* L.) were obtained from the Department of Agriculture Seed Certification Agency, Sangli, Maharashtra, India. Seeds were surface-sterilized with acetone and then used for brief surface sterilization to remove microbial contaminants. Their high volatility and short exposure time prevent penetration into seed tissues, thereby avoiding adverse effects on seed viability and germination, allowing them to be used for germination studies under controlled laboratory conditions. Statistical analysis of the experimental data was performed using IBM SPSS Statistics software, version 26.0 (IBM Corp., Armonk, NY, USA). All quantitative data were expressed as mean ± standard deviation, and statistical significance was evaluated at *p* ≤ 0.05. The seeds of fenugreek that were surface-sterilized with acetone were used for brief surface sterilization to remove microbial contaminants, their high volatility and short exposure time prevent penetration into seed tissues, thereby avoiding adverse effects on seed viability and germination and soaked in water for 2 h, and then pre-soaked seeds were treated with micronutrient-based nanofertilizers in 6 treatments: T_1_ (100 mg/L), T_2_ (200 mg/L), T_3_ (300 mg/L), T_4_ (400 mg/L), T_5_ (500 mg/L), and T_6_ (Control). Readings were taken after 48 h. Number of germinated seeds, Number of non-germinated seeds, Percentage of germination (%), Seedling length, and Seed vigor (Germination Percentage × Seedling Length) were calculated. The impact of all different NPs on crops with respect to seed germination was calculated

### 2.5. Bioassay (Plant Growth) of Nanostructured Zn- and Cu-Doped MnFe_2_O_4_ Fertilizer

The plant growth bioassay was conducted under controlled conditions at Malawadi village (16.86° N, 74.57° E), Bhilwadi, Sangli District, Maharashtra, India, on 20 August 2025. The region experiences a tropical semiarid climate. During the experimental period, the average ambient temperature ranged from 25 to 32 °C, relative humidity was approximately 65 to 75%, and the experiment coincided with the monsoon season, ensuring moderate rainfall and adequate soil moisture. These climatic conditions were conducive to fenugreek growth and were maintained uniformly across all treatments. The experiment was designed to evaluate the effects of Zn- and Cu-doped MnFe_2_O_4_ nanostructured fertilizer on plant growth parameters. Two foliar sprays of Zn- and Cu-doped MnFe_2_O_4_ nanofertilizer were applied to fenugreek plants using six treatment levels. Treatments T_1_–T_5_ corresponded to nanoparticle concentrations of 100, 200, 300, 400, and 500 mg/L, respectively, while T_6_ served as the control and received only distilled water without nanoparticles. Each treatment was conducted in triplicate, where a, b, and c represent three independent biological replicates for each concentration. Thus, treatments such as T_1_-a, T_1_-b, and T_1_-c denote replicate units receiving the same nanoparticle concentration. Fenugreek planting was done on 20 August 2025. The fenugreek seeds were surface-sterilized with acetone and then used for brief surface sterilization to remove microbial contaminants. Their high volatility and short exposure time prevent penetration into seed tissues, thereby avoiding adverse effects on seed viability and germination. Pots were filled with pre-analyzed black cotton soil. Twenty fenugreek seeds were sown per pot at a depth of 2 cm in each pot. Spraying was carried out, and reading was taken seven days after the first application and the second application. The first dose of Zn- and Cu-doped MnFe_2_O_4_ was sprayed on the fourth day of germination, and the second dose of Zn- and Cu-doped MnFe_2_O_4_ was given after 7 days of the first dose. The untreated plants were used as a control. An in vitro experimental setup was arranged in a randomized block design with three replicates. The results of Zn- and Cu-doped MnFe_2_O_4_ nanofertilizer on fenugreek with respect to plant height in (cm), root height (cm), number of leaves, and width of stem (mm) were studied after seven days of first and second applications. Three replicate pots of all treatments were maintained. Measurements were recorded from all plants within each pot, and the mean value per pot was calculated. These pot-wise mean values from three biological replicates were used for statistical analysis. All data are expressed as mean ± standard deviation (SD) of three replicates. Statistical analysis was performed using IBM SPSS Statistics (version 26.0). Differences among treatments were evaluated using one-way ANOVA at (*p* ≤ 0.05).

### 2.6. Bioassay (Chlorophyll Estimation) of Nanostructured Zn- and Cu-Doped MnFe_2_O_4_ Fertilizer

After application of Zn- and Cu-doped MnFe_2_O_4_ nanofertilizer in the fenugreek crop, chlorophyll content in the fenugreek leaf was observed.

The chlorophyll content was measured according to the protocol established [23]. Plant samples treated with Zn- and Cu-doped MnFe_2_O_4_ fertilizer were selected for chlorophyll analysis, while untreated plants served as controls. A leaf sample (1 g) was ground using a mortar and pestle with the addition of 20 mL of acetone, and the mixture was then centrifuged at 5000 rpm for 5 min. The supernatant was recorded using a spectrophotometer (Shimadzu, UV-1900i, Chennai, India) at wavelengths of 645, 652, and 663 nm with 80% acetone used as the blank. Amount of chlorophyll was estimated (milligram chlorophyll per gram of tissue) using the formula

(1) $${\mathrm{Chlorophyll}\;b\;(\mathrm{mg}\;g}^{-1})=\frac{\left[12.7(A_{663})-2.69(A_{645})\right]\times V}{1000\times W}$$

(2) $${\mathrm{Chlorophyll}\;b\;(\mathrm{mg}\;g}^{-1})=\frac{\left[22.9(A_{645})-4.68(A_{663})\right]\times V}{1000\times W}$$

(3) $${\mathrm{Total}\;\mathrm{Chlorophyll}\;(\mathrm{mg}\;g}^{-1})=\mathrm{Chlorophyll}\;a+\mathrm{Chlorophyll}\;b$$

where

A—Absorbance at specific wavelengths.
V—Final volume of chlorophyll extract in 80% acetone.
W—Weight of the fresh tissue extracted.

## 3. Result and Discussion

### 3.1. X-Ray Diffraction Spectroscopy

**Figure 1:**
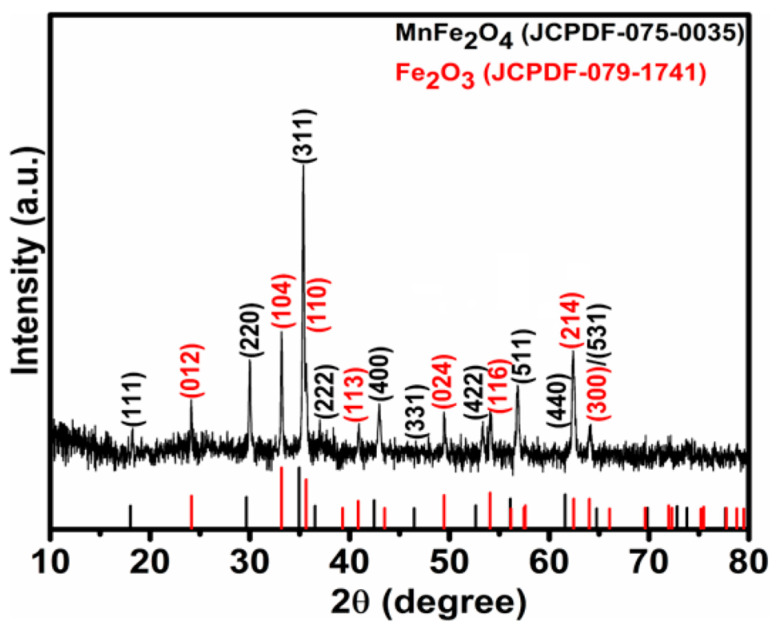
XRD of the synthesized Zn- and Cu-doped MnFe_2_O_4_ nanoparticle.

X-ray diffraction analysis was employed to confirm the phase formation, crystallinity, and structural characteristics of the synthesized Zn- and Cu-doped MnFe_2_O_4_ nanoparticles. The XRD pattern recorded using a Bruker AXS D8 Advance diffractometer with Cu-Kα radiation (λ = 1.5406 Å) exhibits well-defined diffraction peaks at 2θ values of approximately 25°, 30°, 34°, 35°, 43.5°, 50°, 57°, 63°, and 65°, which can be indexed to the crystallographic planes (220), (100), (002), (311), (111), (511), (440), (112), and (310), respectively (Figure 1). These reflections are in good agreement with the standard spinel cubic structure of MnFe_2_O_4_ (JCPDS/ICDD card No. 75-0034), confirming the successful formation of the ferrite phase. Formation of Fe_2_O_3_ was also detected in the X-ray diffraction pattern, and its patterns are well-matched with the JCPDS card number 079-1741.

The absence of additional impurity peaks corresponding to secondary oxide phases indicates that Cu^2+^ and Zn^2+^ ions are effectively incorporated into the MnFe_2_O_4_ lattice rather than forming separate crystalline phases. This suggests successful substitution of divalent cations at the octahedral and tetrahedral sites of the spinel structure, consistent with previous reports on doped ferrite nanoparticles. Minor variations in peak intensity and broadening observed in the doped samples can be attributed to lattice distortion induced by the difference in ionic radii of Cu^2+^ (0.73 Å) and Zn^2+^ (0.74 Å) compared to Mn^2+^ (0.80 Å), which alters the local crystal environment and defect density. The average crystallite size (D) of the Zn- and Cu-doped MnFe_2_O_4_ nanoparticles was estimated using the Debye–Scherrer Equation (4):

(4) $$D=\frac{0.9\lambda }{\beta \cos\theta }$$

where λ is the wavelength of Cu-Kα radiation (1.5406 Å), β is the full width at half maximum (FWHM) of the most intense diffraction peak, and θ is the corresponding Bragg angle. The calculated average crystallite size was approximately 27.01 nm, indicating the formation of nanocrystalline ferrite particles.

The nanoscale crystallite size is particularly advantageous for agricultural applications, as reduced particle dimensions enhance surface area and reactivity, thereby facilitating improved nutrient availability and interaction with plant root systems. Furthermore, the preservation of the spinel structure after dual metal doping highlights the structural stability of MnFe_2_O_4_, making it a promising candidate for multi-micronutrient nanofertilizer formulations. The structure and phase purity of MnFe_2_O_4_ nanoparticles are specifically reported by [24].

### 3.2. Scanning Electron Microscope (SEM)

**Figure 2:**
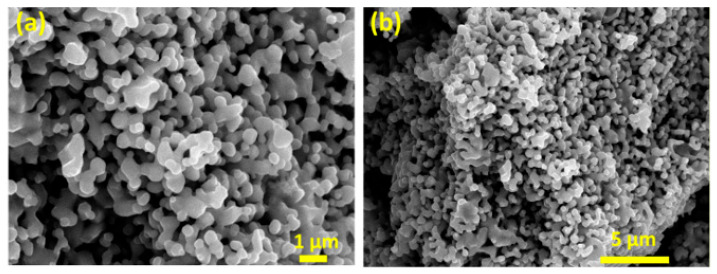
Scanning electron microscopic morphology of Zn- and Cu-doped MnFe_2_O_4_ nanoparticles at. (**a**) 1 μm; (**b**) 5 μm.

To study the surface morphology and to calculate the grain size of the prepared powder. The SEM analysis was carried out to examine the size distribution, morphology, and surface characteristics of Zn- and Cu-doped MnFe_2_O_4_ nanoparticles. These nanoparticles were synthesized using a specific method, and their grain sizes were measured within a 1 μm area to assess their uniformity and structural properties. The SEM analysis of Zn- and Cu-doped MnFe_2_O_4_ nanoparticles revealed a grain size distribution ranging from 93.5 nm. While the particles displayed a range of sizes and some aggregation, they may still be suitable for various applications, particularly those where moderate size variability and aggregation can be tolerated, such as in catalysis or magnetic applications (Figure 2).

### 3.3. Energy Dispersive X-Ray Analysis (EDAX)

**Figure 3:**
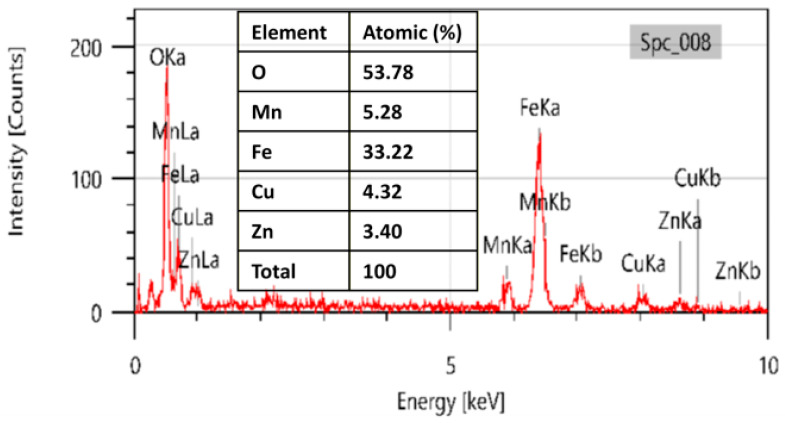
EDAX of synthesized Zn- and Cu-doped MnFe_2_O_4_ nanoparticles.

Energy dispersive X-ray spectroscopy (EDS) was employed to investigate the element composition and verify the successful incorporation of Zn and Cu dopants into the MnFe_2_O_4_ lattice. The EDS spectra were acquired using a JEOL JSM-IT200 scanning electron microscope equipped with an EDAX detector, operated at an accelerating voltage of 20 kV, a working distance of 5 mm, a magnification of 500×, and an acquisition time of 30 s (Figure 3). The EDS spectrum confirms the presence of manganese (Mn), iron (Fe), zinc (Zn), copper (Cu), and oxygen (O), consistent with the targeted doped ferrite composition. Quantitative elemental analysis revealed weight percentages of 6.35% Zn, 7.83% Cu, 8.28% Mn, 52.97% Fe, and 24.57% O. The relative elemental distribution closely aligns with the nominal precursor ratios used during synthesis, indicating effective incorporation of Zn^2+^ and Cu^2+^ ions into the manganese ferrite matrix. The absence of extraneous elemental signals confirms the high chemical purity of the synthesized nanoparticles and suggests that no secondary phases or residual precursor contaminants were formed during the sol–gel process.

### 3.4. Dynamic Light Scattering (DLS)

Dynamic light scattering (DLS) analysis was performed to evaluate the hydrodynamic particle size distribution and colloidal stability of the Zn- and Cu-doped MnFe_2_O_4_ nanoparticles dispersed in aqueous medium. The DLS results indicate a Z-average hydrodynamic diameter of 82.75 nm, confirming the nanoscale nature of the synthesized particles. The particle size distribution by number shows a single dominant peak (Peak 1) centered at 20.31 nm, accounting for 100% of the particle population, which suggests a relatively uniform size distribution without secondary populations. The polydispersity index (PDI) value of 0.260 indicates moderate polydispersity and acceptable dispersion stability, which is characteristic of doped ferrite nanoparticles synthesized via wet chemical routes. The standard deviation associated with the primary peak was 6.981 nm, further supporting the narrow size distribution. The high intercept value (0.890) and overall result quality, rated as “Good,” confirm the reliability of the DLS measurement.

**Figure 4:**
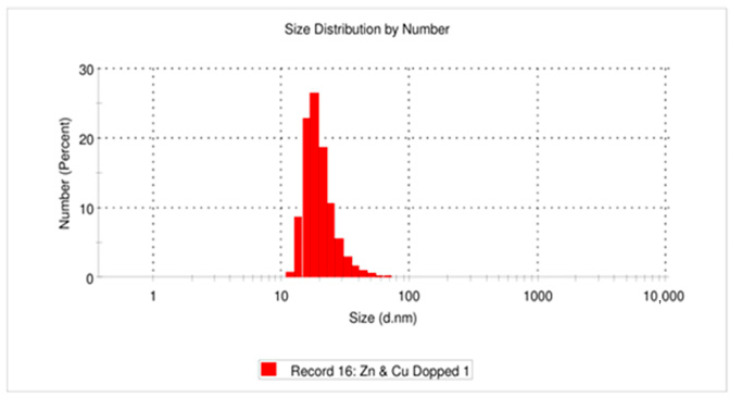
Dynamic light scattering of synthesized Zn- and Cu-doped MnFe_2_O_4_ nanoparticles.

The larger Z-average size compared to the crystallite size obtained from XRD analysis can be attributed to the hydrodynamic diameter, which includes solvent layers and possible minor agglomeration in the suspension. Nevertheless, the observed particle size range is well suited for nanofertilizer applications, as nanoscale dimensions facilitate enhanced surface area, improved dispersion, and efficient interaction with plant tissues. The DLS results confirm that Zn- and Cu-doped MnFe_2_O_4_ nanoparticles exhibit suitable nanoscale dimensions and colloidal stability, supporting their potential application as efficient multi-micronutrient nanofertilizer (Figure 4).

### 3.5. Bioassay of Zn- and Cu-Doped MnFe_2_O_4_ Nanofertilizer on Fenugreek Seed Germination

**Figure 5:**
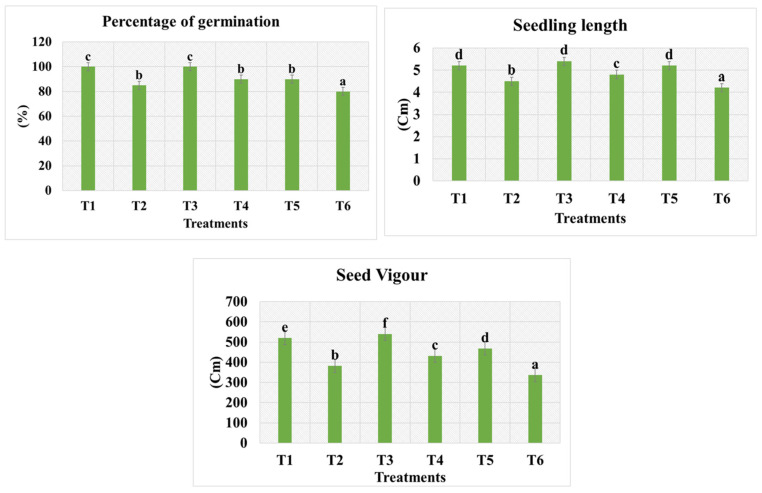
Effect of Zn- and Cu-doped MnFe_2_O_4_ on fenugreek seed germination percentage and seed vigor after two days of treatment. Values are expressed as mean ± SE of three replicates; a, b, c, d, e and f indicate statistically significant differences (*p* < 0.05) according to DMRT. Mean ± SE of three replicates of percentage of germination, seedling length, and seed vigor shows statistically significant differences (*p* < 0.05).

**Figure 6:**
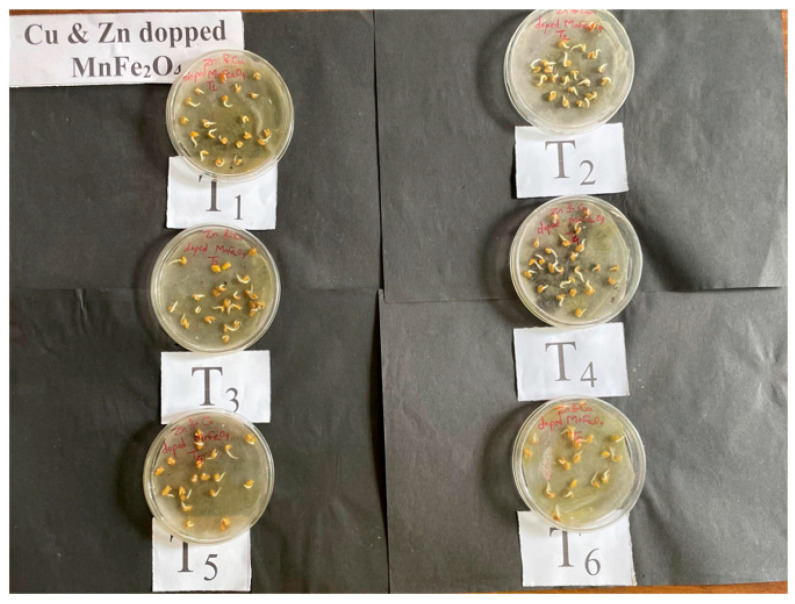
Photograph of the treatment of Zn- and Cu-doped MnFe_2_O_4_ multinutrient nanofertilizer on fenugreek seed germination.

The effect of Zn- and Cu-doped MnFe_2_O_4_ multinutrient nanofertilizer at different concentrations on seed germination, seedling length, and seed vigor in fenugreek was evaluated. The T_1_ and T_3_ treatments showed 100 ± 0.0% germination (Figure 5 and Figure 6), indicating the optimal effect of Zn- and Cu-doped MnFe_2_O_4_ nanofertilizer at these concentrations. The T_4_ and T_5_ treatments achieved a 90 ± 0.5% germination rate, while the T_6_ group showed the lowest germination percentage at 80 ± 1.1%. The highest seedling length was observed in the T_3_ (5.4 ± 0.1 cm), T_1_ (5.2 ± 0.1 cm), and T_5_ (5.2 ± 0.2 cm) treatments, while the T_4_ treatment had a slightly lower seedling length of 4.8 ± 0.05 cm. The T_6_ had the lowest seedling length of 4.2 ± 0.1 cm, demonstrating better root and shoot development with the nanofertilizer treatments. Seed vigor was highest in the T_3_ treatment (540 ± 0.5 cm), followed by the T_1_ treatment (520 ± 0.5 cm). The T_5_ and T_2_ treatments showed vigor values of 468 ± 0.5 cm and 382 ± 0.0 cm, respectively. The T_4_ treatment had a vigor of 432 ± 0.0 cm, and the T_6_ had the lowest vigor of 336 ± 0.5 cm, indicating the positive effect of Zn- and Cu-doped MnFe_2_O_4_ nanofertilizer on seed health and growth. The results indicate that Zn- and Cu-doped MnFe_2_O_4_ multinutrient nanofertilizer significantly enhances seed germination, seedling length, and seed vigor in fenugreek. The T_3_ treatment was the most effective, showing 100% germination. The T_1_ treatment also demonstrated excellent performance in seed vigor and seedling length development. The T_6_ exhibited lower performance across all measured parameters, emphasizing the beneficial effects of Zn- and Cu-doped MnFe_2_O_4_ nanofertilizer. Similar results with iron-based materials on mung bean seed germination have been recorded in previous studies [25]. Plants absorbed nanofertilizer through gradual and continuous release, resulting in stronger seedling growth [26].

### 3.6. Bioassay of Zn- and Cu-Doped MnFe_2_O_4_ Nanofertilizer on Fenugreek Growth Performance After First and Second Spraying

**Figure 7:**
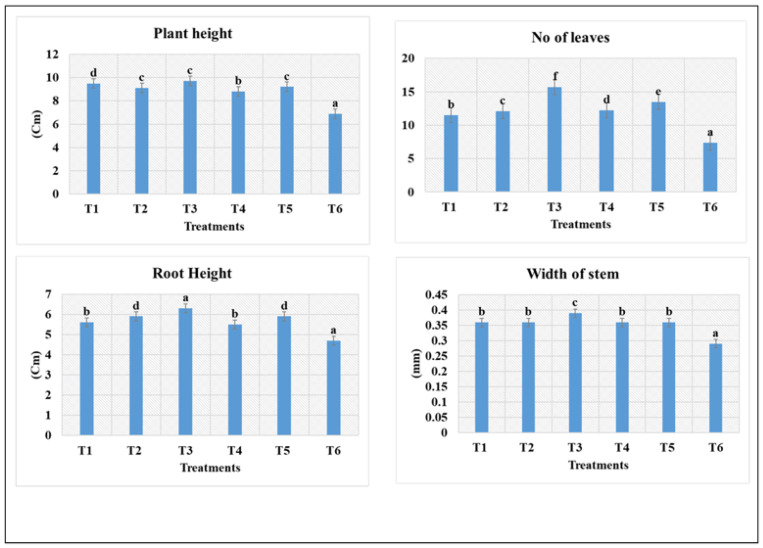
Effect of Zn- and Cu-doped MnFe_2_O_4_ nanofertilizer in fenugreek crop after first spraying for different treatments. Values are expressed as mean ± SE of three replicates; a, b, c, d, e, and f indicate statistically significant differences (*p* < 0.05) according to DMRT.

Application of Zn- and Cu-doped MnFe_2_O_4_ nanofertilizer resulted in notable variations across all growth parameters in fenugreek. Plant height increased consistently from T_1_ through T_4_, with T_3_ achieving the maximum shoot elongation of 9.7 ± 0.4 cm, while the control (T_6_-6.9 ± 0.4 cm) recorded the lowest values. Root height followed a similar trend, where T_3_ and T_5_ (6.3 ± 0.3 cm and 5.9 ± 0.2 cm) produced the longest and most robust roots, indicating enhanced nutrient uptake efficiency. The number of leaves was highest in T_3_-15.7 ± 1.3, followed by T_4_-13.8 ± 0.9, reflecting improved vegetative vigor and photosynthetic potential. Stem width also showed significant enhancement under nanoparticle treatments, with T_3_-0.39 ± 0.02 mm and T_4_-0.36 ± 0.02 mm producing thicker stems than both lower and higher concentrations. In contrast, T_5_ (500 mg/L) exhibited a slight decline in performance compared to the optimal treatments, suggesting potential nutrient imbalance or stress at higher nanoparticle levels. Overall, the mid-range concentrations (300–400 mg/L) demonstrated superior growth responses across all measured parameters (Figure 7). The superior performance at T_3_ suggests that 300 mg/L provides an optimal balance of micronutrient bioavailability without inducing toxicity or oxidative stress. The notable decline in T_5_ across all parameters may indicate nanoparticle overaccumulation and possible phytotoxic effects, which have been reported in high-concentration nano metal oxide treatments. Enhanced root height at T_3_ and T_4_ indicates improved nutrient uptake due to increased root branching and physiological stimulation by metal ions. The results align with reports that moderate nanoparticle dosages improve plant growth by enhancing nutrient uptake efficiency, boosting photosynthesis, and modulating growth hormone pathways. These findings support the potential of doped ferrite nanomaterials as efficient nano-enabled fertilizers for sustainable agriculture.

**Figure 8:**
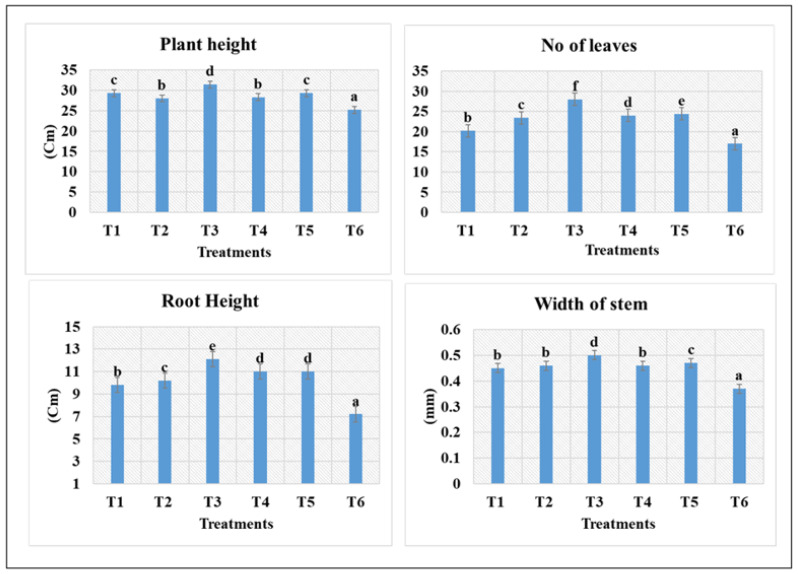
Effect of Zn- and Cu-doped MnFe_2_O_4_ nanofertilizer in fenugreek crop after second spraying for different treatments. Values are expressed as mean ± SE of three replicates; a, b, c, d, e, and f indicate statistically significant differences (*p* < 0.05) according to DMRT.

After the second application of Zn- and Cu-doped MnFe_2_O_4_ nanofertilizer, a notable improvement in the growth performance of fenugreek was observed compared to the control. Among the different concentrations, the 300 mg/L treatment (T_3_) consistently showed the highest enhancement across most growth parameters. Plant height increased markedly under T_3_-31.4 ± 1.1 cm, followed by T_2_-28 ± 1.7 cm and T_5_-29.1 ± 0.8 cm, whereas the control (T_6_-25.2 ± 1.5 cm) exhibited the lowest height (Figure 8). A similar trend was observed in the number of leaves, with T_3_-28 ± 2.1 producing the maximum leaf count, indicating improved vegetative development at moderate nanoparticle concentration. T_3_ showed the highest root height (12 ± 0.5 cm) after the second application of Zn- and Cu-doped MnFe_2_O_4_ nanofertilizer. Stem width also increased significantly in T_3_-0.5 ± 0.3 mm and T_4_-0.4 ± 0.3 mm, suggesting stronger structural growth in treated plants. In contrast, root length did not follow the same pattern; the control plants recorded the highest root length, while all nanoparticle treatments showed comparatively shorter roots, indicating a shift in biomass allocation toward shoot development. Higher concentrations, particularly 500 mg/L (T_5_), showed a slight decline in growth-promoting effects, suggesting potential stress or reduced nutrient efficiency at elevated nanoparticle levels. Overall, the results demonstrate that Zn- and Cu-doped MnFe_2_O_4_ nanofertilizer enhances fenugreek growth, with 300 mg identified as the optimal concentration for maximizing shoot-related parameters. These findings are consistent with earlier reports demonstrating the beneficial role of ferrite-based nanofertilizer in crop growth. A significant enhancement in wheat growth following MnFe_2_O_4_ nanofertilizer application was previously reported [27], supporting the positive physiological role of Mn and Fe synergism. Similarly, higher concentrations of ZnO nanoparticles were shown to increase growth parameters in fenugreek [28], which aligns with the enhanced leaf number and shoot elongation observed in the present study under Zn-containing ferrite treatments. Iron oxide nanoparticles have also been reported to significantly improve shoot length, leaf traits, and root development in mulberry plants, as well as in maize, barley, and wheat [29]. The improved root length observed in T_3_ and T_4_ treatments in this study corroborates these findings and indicates improved nutrient uptake efficiency mediated by iron-based nanostructures. Furthermore, the increasing importance of Mn and Fe in regulating root activity and enzymatic processes has been well documented [16], which may explain the enhanced root growth and overall plant vigor observed under optimal nanoparticle concentrations. The statistically significant improvements recorded at T_3_ and T_4_ confirm that Zn- and Cu-doped MnFe_2_O_4_ nanofertilizer act as efficient multi-micronutrient delivery systems. Their nanoscale size, combined with the synergistic roles of Mn, Fe, Zn, and Cu, enhances nutrient uptake, photosynthetic efficiency, and vegetative growth, highlighting their strong potential for sustainable agricultural applications.

**Figure 9:**
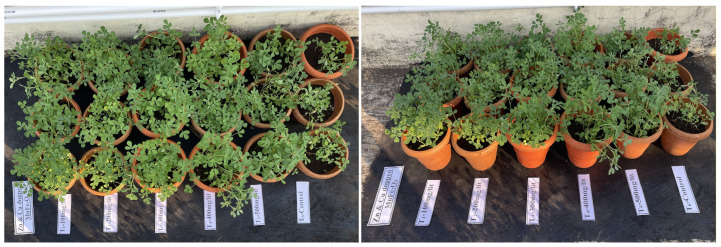
Photographs of the fenugreek plants from different angles after application of the Zn- and Cu-doped MnFe_2_O_4_ nanofertilizer.

The different-angle photograph of the fenugreek plants at different treatments (T_1_–T_6_) after the second spray is illustrated in Figure 9.

### 3.7. Zn- and Cu-Doped MnFe_2_O_4_ Treated Fenugreek Crop Biomass After Harvesting

**Figure 10:**
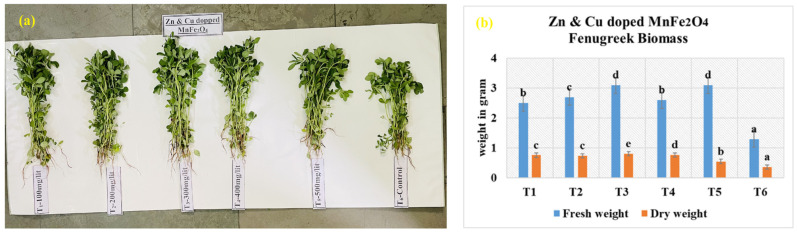
After harvesting analysis, (**a**) a photograph of Zn- and Cu-doped MnFe_2_O_4_-treated fenugreek crop after harvesting and (**b**) its biomass analysis. Values are expressed as mean ± SE of three replicates; a, b, c, d, and e indicate statistically significant differences (*p* < 0.05) according to DMRT.

Application of Zn- and Cu-doped MnFe_2_O_4_ nanofertilizer significantly enhanced the biomass accumulation in fenugreek plants compared to the untreated control (Figure 10a,b). Fresh weight increased across all nanoparticle treatments, with the highest value observed at T_3_-Fresh weight-3.1 g and Dry weight-0.81 g, and T_5_-Fresh weight-3.1 g and Dry weight-0.6 g, followed closely by T_4_-Fresh weight-2.6 g and Dry weight-0.77 g. This indicates that moderate to moderately high nanoparticle concentrations improved water retention, cellular turgidity, and overall vegetative growth. The lowest fresh weight was recorded in the control (T_6_-Fresh weight: 1.3 g and Dry weight: 0.46 g), confirming the positive influence of nanoparticle supplementation on biomass production. Dry weight followed a similar trend, with T_3_ exhibiting the greatest increase, suggesting improved tissue development, nutrient assimilation, and structural biomass formation at optimal nanoparticle concentration. Both fresh and dry biomass data demonstrate that Zn- and Cu-doped MnFe_2_O_4_ nanoparticles effectively enhance biomass accumulation in fenugreek, with 300 mg/L (T_3_) emerging as the most effective concentration for maximizing harvestable yield.

### 3.8. Bioassay of Zn- and Cu-Doped MnFe_2_O_4_ Nanofertilizer on Chlorophyll Content in Fenugreek Growth Performance

**Figure 11:**
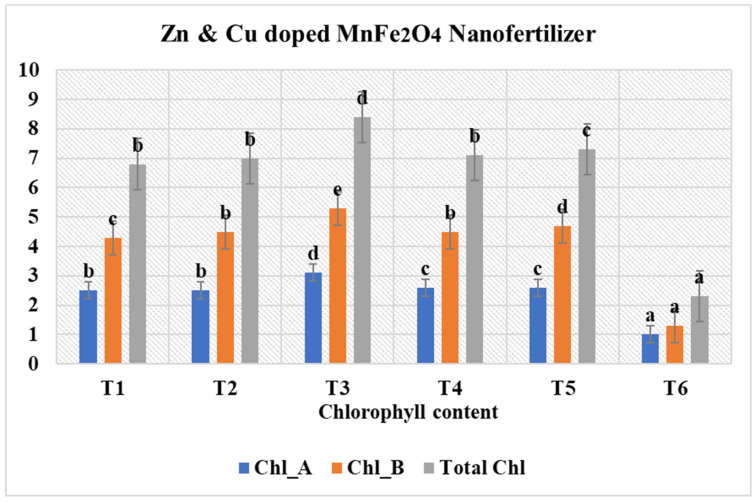
Effect of Zn- and Cu-doped MnFe_2_O_4_ nanofertilizer at different concentrations on chlorophyll content in fenugreek leaves. Values are expressed as mean ± SE of three replicates; a, b, c, d, and e indicate statistically significant differences (*p* < 0.05) according to DMRT.

The T_3_ and T_5_ nanofertilizer concentrations produce the highest chlorophyll content (8.4 mg/g and 7.3 mg/g tissue, respectively), making them the most effective concentrations for enhancing photosynthetic activity in fenugreek leaves. Application of Zn- and Cu-doped MnFe_2_O_4_ nanofertilizer produced a significant, dose-dependent enhancement in chlorophyll content of fenugreek plants compared with the untreated control (T_6_-2.3 mg/g). Chlorophyll A and chlorophyll B levels increased progressively from T_1_ to T_3_, with T_3_ exhibiting the highest pigment accumulation. This treatment showed the greatest concentration of chlorophyll A, chlorophyll B, and total chlorophyll (from Equations (1)–(3)), indicating optimal photosynthetic efficiency at 300 mg/L. T_4_ (400 mg/L) also maintained high chlorophyll content, slightly lower than T_3_ but still markedly higher than T_1_, T_2_, and T_6_. At the highest dose T_5_, chlorophyll levels declined compared with T_3_ and T_4_, suggesting possible stress or pigment degradation at elevated nanoparticle concentrations. The control (T_6_) exhibited the lowest chlorophyll A, chlorophyll B, and total chlorophyll values, highlighting the strong stimulatory effect of Zn- and Cu-doped MnFe_2_O_4_ fertilizer supplementation, and midrange nanofertilizer concentrations T_3_ and T_4_ were theoretically optimal for maximizing chlorophyll biosynthesis and improving photosynthetic performance in fenugreek (Figure 11).

## 4. Conclusions

The study demonstrates the successful application of Zn- and Cu-doped MnFe_2_O_4_ nanofertilizer as an efficient micronutrient delivery system for enhancing fenugreek growth. Among the tested concentrations, T_3_ and T_4_ consistently produced the most pronounced improvements in plant height, root length, leaf number, and stem thickness compared with both lower concentrations and the untreated control (T_6_). In contrast, a higher concentration of T_5_ resulted in a slight decline in growth performance, indicating the importance of dosage optimization. The novelty of this work lies in the combined Zn and Cu doping within a MnFe_2_O_4_ ferrite matrix, which enables the simultaneous and controlled delivery of four essential micronutrients (Zn, Cu, Mn, and Fe) in a single nanostructured formulation. This synergistic approach enhances nutrient use efficiency, promotes balanced physiological processes, and supports healthier crop development without the need for multiple conventional fertilizers. The nanoscale characteristics of the doped ferrite particles, together with their potential antibacterial activity, likely contribute to improved root health and nutrient uptake efficiency. These findings distinguish the present study from earlier reports that primarily focused on single metal or single oxide nanofertilizer; the results establish Zn- and Cu-doped MnFe_2_O_4_ nanofertilizer as a promising, sustainable alternative to conventional micronutrient fertilizers. By reducing excessive fertilizer inputs and improving nutrient bioavailability, this nano-enabled strategy aligns with modern agricultural practices aimed at enhancing crop productivity while minimizing environmental impact.

## Data Availability

The original contributions presented in this study are included in the article. Further inquiries can be directed to the corresponding authors.
